# The Cyclical Return of the IQ Controversy: Revisiting the Lessons of the Resolution on Genetics, Race and Intelligence

**DOI:** 10.1007/s10739-021-09637-6

**Published:** 2021-05-21

**Authors:** Davide Serpico

**Affiliations:** Department of Classics, Philosophy and History, University of Genoa, Via Balbi, 2, 16126 Genoa, Italy

**Keywords:** IQ controversy, Group differences, Behavioral genetics, Intelligence, Academic freedom, Scientific communication

## Abstract

In 1976, the Genetics Society of America (GSA) published a document entitled “Resolution of Genetics, Race, and Intelligence.” This document laid out the Society’s position in the IQ controversy, particularly that on scientific and ethical questions involving the genetics of intellectual differences between human populations. Since the GSA was the largest scientific society of geneticists in the world, many expected the document to be of central importance in settling the controversy. Unfortunately, the Resolution had surprisingly little influence on the discussion. In 1979, William Provine analyzed the possible factors that decreased the impact of the Resolution, among them scientists’ limited understanding of the relationship between science and ethics. Through the analysis of unpublished versions of the Resolution and exchanges between GSA members, I will suggest that the limited impact of the statement likely depended on a shift in the aims of the GSA due to the controversies that surrounded the preparation of the document. Indeed, the demands of the membership made it progressively more impartial in both scientific and political terms, decreasing its potential significance for a wider audience. Notably, the troubled history of the Resolution raises the question of what can make effective or ineffective the communication between scientists and the public—a question with resonance in past and present discussions on topics of social importance.

## Introduction

Since the nineteenth-century beginnings of the debate on *nature* and *nurture*, scholars have disagreed on the source of observed psychological variation within and between groups. Research on human populations, heredity, and intelligence have generated heated discussions with respect to many publications (e.g., Herrnstein 1971; Herrnstein and Murray 1994; Jensen 1969; Levin 1997; Lynn 2008, 2010; Rushton 1995; Wade 2014; see also Weinberg 2020), as well as to specific events (Adams 2019; Lally 2018; Meredith 2018).

On several occasions, scientific societies and groups of scholars have attempted to settle the debate by publishing official statements and petitions, among them UNESCO (1950, 1952), American Association for the Advancement of Science (1963, 1967), American Psychological Association (2001), Cubelli (2010), Letters: “A Troublesome Inheritance” (2014), and American Society of Human Genetics (2018). Such statements have often divulged state-of-the-art knowledge in order to counter racism and discrimination. For instance, the 1950s UNESCO project was motivated by the belief that science had already produced the correct antiracist knowledge and the next step was to promulgate such knowledge worldwide. Nevertheless, the statement—like other documents of the same sort—was unable to achieve its goal (see Brattain 2007; Provine 1986).

Among such statements, the “Resolution on Genetics, Race, and Intelligence” (Russell 1976; hereafter Resolution), published by the Genetics Society of America (GSA), is particularly interesting for its long and convoluted drafting process, which lasted roughly three years and involved many contributors. Indeed, the process required the efforts of hundreds of GSA members as well as an ad hoc Committee on Genetics, Race, Intelligence (1974–1975) appointed by the Society, which consisted of Harrison Echols (University of California, Berkeley), Walter E. Nance (Indiana University), David D. Perkins (Stanford University), Janice B. Spofford (University of Chicago), John R. G. Turner (then at State University of New York Stony Brook), and Elizabeth S. Russell (Jackson Laboratory, Bar Harbor, Maine). On the scientific side, the document discussed methodological limitations of genetic studies of human intelligence. On the ethical side, it discouraged racism and discrimination.

Since the GSA, at the time boasting around 2600 members, was the largest scientific society of geneticists in the world, many expected the document to be of central importance in providing guidance to policymakers and settling the IQ controversy. For instance, Evelyn Witkin, a geneticist at Rutgers University, commented that the Resolution may well have been “the most important statement that this generation of American geneticists is called upon to make, and each of us is obliged to express an honest judgment.”^1^ In the early phases of the project, the intention was to disseminate the Resolution in as many outlets as possible, including publication in *Genetics* (the official journal of the Society), *Science, Nature,* and *Bioscience* as well as through press conferences. In the end, the final version of the document was only published in *Genetics* and had surprisingly little impact on discussions of genetics, race, and intelligence.

In 1979, William Provine, a historian of evolutionary biology and population genetics at Cornell University, analyzed the events relating to the drafting of the Resolution and pointed to factors that decreased its public resonance (Provine 1979; see also Provine 1986).^2^ According to Provine, the statement was not impactful because it did not clearly rebuke the hereditarian hypothesis and was not sufficiently publicized. Moreover, he believed that GSA members were “ethically naïve,” that is, they simply did not see the moral issues at stake (1979, p. 34). He supported this view by noting that, among the many members of the Society, only one addressed the question of the relationship between science and morality, namely, Norman H. Horowitz, a geneticist at the California Institute of Technology (Provine 1979, p. 25; 1986, p. 880).

In 1977, officials of the Genetics Society of America deposited documents pertaining to this debate in the society’s records held at the American Philosophical Society in Philadelphia. However, recently deposited materials at the University of Leeds Library, Special Collections provide an opportunity to investigate the history of the Resolution further.^3^ The collection of the population geneticist John R. G. Turner, School of Biology, University of Leeds, who was a member of the GSA ad hoc Committee, includes dozens of letters from his private correspondence with GSA members as well as several unpublished drafts of the Resolution.^4^ These materials help illuminate the deliberations of the ad hoc Committee and offer a revised perspective over that of Provine.

In the first section of the paper, I focus on the social context in which the IQ controversy originated. This helps clarify why an important scientific society like the Genetics Society of America decided to take a public stand. In the second section, I describe the process of drafting the Resolution and discuss the major conceptual and perspectival differences between the various versions of the document that followed one after another between 1973 and 1976. Through the chronological analysis of correspondence, I highlight what pressures shaped the Resolution and provide a better sense of the development of the GSA’s action. In the third section, I discuss the factors that may have decreased the influence of the document. In contrast to Provine’s conclusions, my analysis of the correspondence suggests that the statement’s minor impact probably had little to do with scientists’ limited appreciation of ethical questions.^5^ An interesting aspect that emerges from the correspondence is that the attempt to meet the demands of the membership made the document progressively more “impartial” and less informative in both scientific and political terms. This probably contributed to the decreased significance of the Resolution for both the public and the GSA members themselves. In this sense, Provine’s hypothesis that the statement had a small impact *because* it was not publicized enough is probably misleading. As I suggest, the “burying” of the Resolution*,* rather than being a cause of its limited impact, might rather reflect a shift in the aims of the GSA as its members became increasingly aware of the complexity of the debate.

In the final section, I ask whether anything could have been done differently. In this sense, the history of the Resolution represents an ideal means for assessing past and contemporary debates on genetics, race, and intelligence. Discussions on this topic have continued to occur periodically despite the past efforts of scientific societies like the GSA. Many issues debated today were already being discussed in the 1970s—including academic freedom, the relationship between science and ethics, and the social implications of genetics findings. This raises the question of what can make the communication between scientists and the public effective or ineffective—a question with resonance in past and present discussions on topics of social importance.

## What Led the GSA to Take a Stand?

The debate on genetics, race, and intelligence has been revived on several occasions in the last century, but four periods were particularly important: (1) in 1916 mental tests were introduced in the US by psychologist Lewis Terman, which supported the eugenics movement by providing the means to analyze human intellectual abilities in quantitative terms (see Daniels 1973; Gould 1981; Kevles 1985; Terman 1916); (2) the early 1950s, when UNESCO published two statements on the subject (UNESCO 1950, 1952; see Bangham 2015; Brattain 2007; Provine 1986); (3) the 1970s, when, as we shall see, the question of group differences became intertwined with heritability research that resulted in the IQ controversy; and (4) following the 1994 publication of Richard Herrnstein and Charles Murray’s *The Bell Curve*, after which the debate arose again (Block 1995; Devlin et al. 1997).

As a précis to the third period, in the late 1960s, the work of psychologists such as Cyril Burt, Arthur Jensen, and Hans Eysenck drew attention to the findings of behavioral genetics and their putative educational implications, causing a firestorm. Jensen’s 1969 paper “How Much Can We Boost Our IQ and Scholastic Achievements?” was particularly instrumental in kindling the discussion. Jensen pointed to the failure of compensatory educational programs and attributed such failure to disparities in IQ, which, he stated, were mostly due to inherited differences among individuals. Moreover, Jensen found reasonable the hypothesis that hereditary factors were involved in IQ differences between human populations.^6^

Jensen’s message was carried to the public by, among others, Harvard professor of psychology Richard Herrnstein and Stanford Nobel Prize winning physicist William Shockley. Herrnstein argued that if intelligence is important in success, and if it is largely heritable, then there will be genetic differences between members of different socioeconomic classes (Herrnstein 1971; for a critical review, see Daniels 1973). Shockley, in turn, claimed that “our nobly intended welfare programs are promoting dysgenics-retrogressive evolution through the disproportionate reproduction of the genetically disadvantaged” (Kevles 1986, p. 271) and urged the National Academy of Sciences to pursue research on the topic.^7^ As Andrew Colman noted, a concept that started out as a “not unreasonable hypothesis” was increasingly presented as factual (2016, p. 187; see also Lewontin 1970).

Jensen’s paper had considerable impact on public discussions on education, especially among policymakers. As Daniels reported, Jensen appeared before various Congressional committees “to deliver his message that money spent on compensatory education programs is ‘lavish’ and ‘extravagant’” (1973, p. 25), with his 1969 inserted study into the Congressional Record. According to Daniel Moynihan (a former member of the Kennedy and Johnson administrations who at the time was serving as an advisor to President Nixon), “the winds of Jensen were gusting through the capital at gale force” (Daniels 1973, p. 25; see also Rose et al. 1984, p. 19). President Nixon’s 1973–1974 budget included a proposal to demolish programs aimed at creating educational equality.

The advent of hereditarianism inspired strong opposition against psychometric and genetic findings. Since most hereditarians were not geneticists by education, some critics focused on how psychologists like Jensen (1969, 1972) and Eysenck (1971) misunderstood genetic data or misused quantitative genetic methods (Cavalli-Sforza and Bodmer 1970; DeFries 1972; Lewontin 1974). Douglas Futuyma, an evolutionary biologist at the State University of New York, illustrated this point:

Jensen and others … have asserted their conviction that races and social groups differ genetically in IQ, without (in my opinion) sufficiently emphasizing to lay audience the limitations on the data, and indeed, the limitations on the capacity of psychometrics and genetics to resolve the problem (given the constraints on experimental design). It is important … to communicate to this audience the extent and importance of these limitations.^8^

Most of the critics, however, pointed at the potentially harmful social implications of hereditarianism (Hirsch 1975; Kamin 1974; Kevles 1985; Paul 1998; Rose et al. 1984). Indeed, hereditarians called into question the principle of equal opportunity, so that scientific and ethical questions became inextricably interconnected (Daniels 1973; Panofsky 2014; Turner 1970).

As Provine noted, this was the period of the Civil Rights Movement: “there was no time in the history of America when thoughtful citizens were less sympathetic to hereditarian explanations for the differential success of racial groups” (1986, pp. 877–878). The controversy was on the front pages of important newspapers and magazines (Bliss 2018; Snyderman and Rothman 1988). For instance, in a 1969 article published in the *New York Times,* Lee Edson coined the term *Jensenism* to denote “the theory that IQ is largely determined by the genes,” a theory that Jerry Hirsch later called “an intellectual disgrace” (Hirsch 1975, p. 25; see also Lewontin 1970). The study of IQ thus became shrouded by an aura of scandal, which brought personal abuse upon several scholars (Flew 1973; Jensen 1972; see also Colman 2016).

A conspicuous group of leading scientists—among them Raymond Cattell, Francis Crick, Hans Eysenck, Richard Herrnstein, Arthur Jensen, and Jacques Monod—signed a petition aimed at encouraging freedom in the study of the genetics of intelligence (Page 1972). On the scientific side, the document claimed that scholars who studied the role of inheritance in human behavior believed that such hereditary influences were very strong. But the central problem identified by the petition was political in nature:

The history of civilization shows many periods when scientific research or teaching was censured, punished, or suppressed for non-scientific reasons, usually for seeming to contradict some religious or political belief. … Today, a similar suppression, censure, punishment, and defamation are being applied against scientists who emphasize the role of heredity in human behavior. Published positions are often misquoted and misrepresented; emotional appeals replace scientific reasoning; arguments are directed against the man rather than against the evidence (e.g., a scientist is called “fascist,” and his arguments are ignored). (Page 1972, p. 660)

It is questionable, however, whether hereditarians’ academic freedom per se was under attack. For instance, Vetta (1973), in response to the petition, did not deny that researchers had the right to investigate the genetics of human behavior, but he criticized the scientific content of the petition. Likewise, most critics were accusing hereditarians of being irresponsible because they were drawing educational and social prescriptions from genetic data, data that might well turn out to be inaccurate. Hirsch, for instance, wrote that “either Jensenists knew what was being perpetrated and [were] therefore responsible, or did not and [were] therefore irresponsible” (1975, p. 27). In sum, the IQ controversy generated a torrent of publications on a variety of questions involving human equality, discrimination, academic freedom, and social policies, as well as the relationship between scientists and society. It was in this context that the Genetics Society of America decided to take a public stand.

Below, I describe the process that gave rise to the Resolution, divided into three phases. In phase one, the committee produced five different drafts; in the second and third phases, three versions of the statement were disseminated among the whole GSA membership. As will be clear, correspondence between GSA members was of vital importance to the revision of the Resolution and its final form. Throughout the process, I argue, the document became progressively more impartial and less informative, contributing to decrease its potential significance for the public and hence diluting its ability to serve as an authoritative guide to the debate for the general public.

## Phase One: A Somewhat Hesitant Start

The 42nd annual meeting of the GSA, held in Berkeley, California in August 1973 (concurrently with the 13th International Congress of Genetics), included a session on “Genetics, Race and IQ” in which biologists, psychologists, and historians pointed out major difficulties in assessing the genetic contribution to IQ group differences. The general consensus at the meeting was that IQ tests would not achieve equally valid results for different cultural groups.

At the Business Meeting of the society, Harrison Echols, a professor of molecular and cell biology at the University of California, Berkeley, presented a petition that harshly criticized hereditarianism and solicited signatures. The petition statement was not, however, approved by the membership; as Provine explained (1979), the consensus was that it was too strong.^9^ However, there was a motion from the floor that the Society’s executive committee appoint an ad hoc committee to draft a statement expressing the official position of the Society in the controversy. The statement was to be submitted to the membership for approval and published in some form.

To get a clearer sense of the controversy among members, it is worth considering the following extract from the minutes of the Business Meeting laying out the charge for the ad hoc committee:

An extended discussion endued over a proposed resolution concerning the Society's position on the subject of Genetics, Race and Intelligence. It was moved and seconded that the petition be referred to the Committee on Public Relations for dissemination to the membership. Signatures returned would be retained for distribution to appropriate organizations or to the mass media. During the discussion an amendment to the motion was proposed and seconded to change the wording in the resolution somewhat. Following discussion, the amendment passed by a narrow margin. During further discussion of the amended motion a motion was made from the floor to table it. This motion passed.

Then a resolution was proposed and seconded from the floor that the executive committee be asked to appoint a committee to draft a resolution on the subject of Genetics, Race and Intelligence to be sent to the membership in the form of a ballot. The proposer indicated his intent that this be an ad hoc committee and not the Committee on Public Relations. Brief discussion followed and the resolution was passed by an overwhelming majority.^10^

Accordingly, the Society’s president, Melvin Green, appointed members of the Committee, drawing from a variety of areas of expertise.^11^ The Committee on Genetics, Race, Intelligence originally consisted of Echols along with Walter E. Nance (human geneticist, Indiana University), David D. Perkins (*Neurospera* geneticist, Stanford University), Janice B. Spofford (evolutionary biologist, University of Chicago), John R. G. Turner (population geneticist, State University of New York Stony Brook), and Elizabeth S. (“Tibby”) Russell (mammalian geneticist, Jackson Laboratory, Bar Harbor, Maine), who served as chairperson.^12^ After Perkins resigned in August 1975, he was replaced by James F. Crow (population geneticist, University of Wiscon-Madison). The aim of the statement they were charged to formulate was to advise the non-academic community on how to interpret scientific findings. As in the case of UNESCO’s project of the 1950s, the GSA’s action was premised on the belief that it was possible to amend racism by disseminating scientific knowledge about the “true” relation between genetics, race, and intelligence.^13^

### The Early Drafts of the Resolution

In the drafting of the Resolution, the committee proceeded sequentially through five different versions of the document. All of the drafts were written by Elizabeth Russell and then shared with members of the committee. Here I focus on three early versions of the Resolution, namely, Proposed Position Paper, Draft #1, and Draft #4, all of which are available in the Turner Collection at Leeds but are not in the GSA Records at the American Philosophical Society.^14^

The first version of the Resolution, entitled “Proposed Position Paper” (hereafter PPP), called on geneticists to oppose unsound scientific and social practices in order to avoid a revival of racism. PPP held that the silence of geneticists who opposed discriminatory policies was one of the reasons why such policies were able to be implemented. As in the case of Echols’s petition, which inspired PPP, this first version of the Resolution gave voice to the environmental side of the controversy. Indeed, the document was sharply critical of hereditarianism:

In recent years there has been a revival of theories which purport to show inherited differences in intelligence between races and social classes. On the basis of these theories, their proponents have suggested changes in social policy ranging from school segregation to sterilization of the “unfit.” The history of earlier eugenics movements demonstrates that great social damage can result from applications of faulty scientific reasoning. Unfounded theories of race improvement were used in the first part of the 20th century as the “scientific” basis for sterilization laws in 31 states, miscegenation laws, and racially restrictive immigration laws.^15^

On the scientific side, PPP criticized the hereditarian theory on three separate questions: 1) whether IQ scores are an adequate measure of intelligence; 2) whether IQ is largely heritable; and 3) whether racial and social class differences in IQ are inherited. Thus, the document concluded, “THERE IS NO CONVINCING EVIDENCE OF GENETIC DIFFERENCE IN INTELLIGENCE BETWEEN RACES” (capitals in the original).^16^

On the ethical side, PPP claimed that social policies proposed on the basis of hereditarian theories were unwarranted; rather, every child must be considered as a valuable individual rather than as a member of a particular racial or socioeconomic group. This was a vital point, given the frequent misinterpretation of statements about characteristics of populations. As Turner noted, “any statement about averages, whether right or wrong, becomes translated in the public mind as a statement about all members of the averaged group.”^17^

In January 1974, Russell sent the first draft of the Resolution to the committee, revised according to the suggestions she had received. Draft #1 is far more neutral than its precursor: both the political and scientific claims were significantly weakened, and there is no explicit criticism of hereditarian positions. Citations from hereditarians such as Jensen were included to summarize the hereditarian view without bias. A disclaimer on freedom of inquiry was also inserted:

We have no desire to discourage research on human intelligence. Freedom of scientific inquiry is a basic tenet of western society, and our knowledge of the human mind is very incomplete.… But we reject simplistic answers to complex questions.^18^

On the scientific side, the committee agreed that genetic differences between individuals—in appropriately selected, culturally homogeneous groups—may account for approximately 80 percent of the observed variation in the aspects of intelligence measured by IQ tests, as heritability analyses seemed to attest (see Eysenck and Kamin 1981). However, Draft #1 stressed that the “undisputed fact” of low average scores on IQ tests in some groups does not in any way prove their genetic inferiority:

Our concern as geneticists is that neither theory nor practice in education or politics shall rest upon a premise of difference in mental capacity between races *unless or until the reality of such a difference has been established.* (emphasis added)

Later, the relationship between ethical and empirical questions generated discussions among the membership. Indeed, the claim cited above seems to imply that political equality should depend on genetic equality, which in turn might imply that, if we find evidence of a genetic difference between groups, we may treat people in the groups differently. Thus, later versions of the Resolution did not draw such a problematic inference, but the uncertain relationship between factual and normative aspects of the controversy is probably one reason why the GSA committee often kept scientific and political questions separate from each other (as will be discussed below).

Between February and October 1974, the committee produced three further drafts, only one of which is extant.^19^ For the present discussion, however, Draft #4 well illustrates how the various versions gradually adopted a more cautious tone that substantially departs from from PPP*.*^20^ The new version brought back criticism of hereditarians and omitted direct citations from their works. This generated controversy within the committee; for example, Turner urged Russell to restore quotations from hereditarians. Another point of divergence lay in the scientific thesis; as a result, four different paragraphs (written by Echols, Nance, Perkins, and Russell, respectively) were included in Draft #4 about the extent to which geneticists disagreed on heritability data.^21^

To summarize, in the first phase, the committee swung from a Resolution supporting one side of the controversy to a neutral one and then back again to the original position. Draft #4 then served as the basis for the first official version of the Resolution, which was disseminated to all the Society’s members.

## Phase Two: Tensions in the Genetics Society of America

In January 1975, the first official version of the Resolution (hereafter Statement #1) was sent to the GSA membership for approval. The contents of this document included no major changes from Draft #4. It was divided into four separate sections: 1) preamble, 2) scientific statement, 3) implications for society, and 4) the role of geneticists.^22^

GSA members were asked to respond to each subsection as to whether they agreed or disagreed with the substance, felt insufficiently informed to judge, or were against taking a stand. Between January and February 1975, the committee received the membership’s responses by mail, with close to half of the GSA membership responding to the poll (1099 members in total). A great majority (almost 90 percent) agreed with the substance of each of the four sections (see Table 1).^23^

**Table 1:** Results of the poll on the first official version of the “Resolution of Genetics, Race and Intelligence.” John R. G. Turner Collection, University of Leeds Library, Special Collections (MS 2044)

Section	Agree with substance	Disagree with substance	Insufficiently informed	Against taking stand
Preamble	975 (88.7%)	76	18	50
Statement	972 (88.4%)	76	24	54
Implications	1056 (96.1%)	14	3	40
Role	1041 (94.8%)	24	7	42

In the following months, the committee received about eighty-five letters with comments on Statement #1. Some were wholly supportive, many were generally favorable but suggested specific alterations, and many others were sharply critical. Three types of problems were cited by the members who opposed the Resolution: (1) the scientific validity of the statement; (2) the political nature of the GSA’s action or the political implications of the text’s tone; and (3) the conflation of ethical and empirical questions. In the next section, I analyze the major controversies that shrouded Statement #1 and were decisive for driving the Resolution towards impartiality.

### Dissenting Voices on Statement #1

Regarding the scientific validity of the statement, the rejection of psychometric and heritability studies disturbed many scholars, including Giuseppe Bertani, Everett Dempster, William Libby, Newton Morton, Eliot Spiess, and Sewall Wright. Some believed that IQ was a good—though imperfect—measure of intelligence. Others argued that evidence existed for a sizable genetic contribution to IQ differences within and between groups—or, at least, that there was no definitive evidence for the contrary assumption. For instance, Earl Green, a geneticist at Jackson Laboratory, Bar Harbor, Maine (and a colleague of Russell’s), criticized the committee for having overestimated current knowledge on the topic:

In the face of the scanty evidence to date, it seems fatuous to say there is NO CONVINCING EVIDENCE OF GENETIC DIFFERENCE IN INTELLIGENCE BETWEEN RACES. One could say the same thing when there is no evidence of any sort at all.^24^ (capitals in original)

Moreover, Green noted that the sentence in capitals might allow the interpretation that evidence was abundant and negative. Biologist Robert Fowler, then at the University of San Francisco, was more adamant in his conclusions, arguing that one could not deny that intelligence was predominantly determined by genetic factors, because this would be contrary to fundamental principles of genetics:

[This] is not merely incorrect; it is ridiculous for a geneticist. This implies that the formation of the human brain and the remaining components of the nervous system, which are responsible for the expression of behavior termed intelligence, have little genetic basis. It also implies that the difference in intelligence between humans and any other animal, for example, a frog, is due almost entirely to our different environments.^25^

It is worth noting that neither hereditarians nor those supporting environmental influences had (or have) ever denied the importance of the genotype in cognitive differences *between different species*. Moreover, the existence of *some* genetic basis for human intelligence was not being questioned. Although the details of the controversy have slightly changed over time (Paul 1998), the disagreement has mostly resided in the suitability of heritability studies to assess the relative magnitude of genetic and environmental influences on cognitive differences *within* the human species.

Fowler interpreted heritability data as capable of explaining some things regarding the way that the genotype determines the phenotype, for example, how genes cause the development of different brain functions and structures in different species. However, it should be noted that the relationship between heritability data and genetic causality is not at all straightforward. I will return to this, since this aspect emerges more clearly later in the correspondence.

Regarding the political nature of the GSA’s action, many scholars (for example, Giuseppe Bertani, James Crow, John DeFries, Everett Dempster, Earl Green, and Sewall Wright) argued that the GSA, as a scientific society, should have concerned itself with science only, since scientists are not more qualified than others to express ethical and political opinions. Moreover, Dempster, William Libby, E. Python, and Benjamin Rasmusen described Statement #1 as politically biased for its attack on unnamed individuals. In this view, connecting hereditarianism to eugenics and Nazism was perceived as unfair towards the view of hereditarians.^26^

Others addressed ethical questions involved. For example, Bernard Davis, Norman Horowitz, and John Turner criticized Statement #1 for being unable to disentangle moral problems from scientific ones. They felt that the document placed the GSA in a particularly weak moral position because it implied that egalitarianism (an ethical stance) should depend on the genetic equality of all humans (a factual statement). For instance, according to Davis, a biologist at Harvard Medical School:

Wide publicity, and much governmental support, has been given to the view that equality of opportunity is best measured by ethnic parity in the distribution of all kinds of jobs and school admissions. People are entitled to believe in such parity as a moral imperative. However, if they believe in it as a consequence of an assumption about the distribution of genetic potential, that assumption is subject to scientific scrutiny.

Moreover, Davis also compared the draft Resolution with the 1963 report of the American Association of the Advancement of Science (AAAS 1963), which considered the use of evidence for race differences as a basis for defending segregation:

The Committee could clearly point out that the evidence did not support the conclusion and was irrelevant, since the Supreme Court had ordered equal civil rights primarily on grounds of moral principle rather than of assumed or demonstrated intellectual equality.^27^

On this ethical problem, Turner also commented: “To say that racial discrimination is wrong because there is no genetic difference is to invite the counter-proposition that racial discrimination is right because there is a genetic difference.”^28^

To summarize, many GSA members criticized the implicit attempt of Statement #1 to favor one side of the controversy. This persuaded Oliver Smithies (University of Wisconsin geneticist and president of GSA, 1975–1976) to continue the process: he made a motion for drafting an alternative version of the Resolution (hereafter Statement #2), analyzed in the next section.

## Phase Three: Towards Impartiality

Based on a simple numerical accounting—the approval of around 90 percent of members to all sections of Statement #1—the GSA’s course of action in the winter of 1975 seemed straightforward: to publish the Resolution as formulated along with a tabulation of the responses from the members. However, in March, Smithies proposed not to publish Statement #1 and drafting an alternative document that addressed the concerns expressed by members. Arguing that the publication of Statement #1 would be a serious failure for the GSA, Smithies stated that, in his view, “a vital part of all scientific endeavors is the ability to accept criticisms and to attempt to answer them.”^29^ Thus, Smithies himself prepared a new version aimed at reflecting the scientific ideas that emerged from the letters and at creating a better separation of scientific facts and ethical principles.

### The Alternative Version of the Resolution

A limitation that Smithies identified in Statement #1 was the emphasis on the poor evidence for genetic differences in intelligence between human populations. Such emphasis, in his view, concealed the equally valid proposition that there was no convincing evidence that such differences were environmental in origin. For this reason, Statement #2 recognized how technically problematic the issue was:

A genetic component for IQ differences within a group does not necessarily imply the existence of a significant genetic component in IQ differences between groups: there may be overriding effects caused by differences in their environments. The problems of unravelling the various factors are so great that it is not possible at present to say unequivocally that there is or is not an appreciable genetic component in differences in IQ between racial groups in this country.^30^

This version was also considerably more balanced than the previous one in terms of the political connotations of the debate. In order to decrease the bias against hereditarians, the statement addressed criticisms as well towards those who believed that environmental factors played a significant role:

The excesses of the early eugenics movement, despite the idealistic intentions of its founders, show the pitfall of naive hereditarian assumptions. Equally unsupportable is a doctrinaire environmentalism that denies any significant role of heredity in important human behavioral traits. The fact that prejudiced misuses of genetic ideas can have tragic consequences when converted into national policies is exemplified by the hereditarian policies of Nazi Germany and the Lysenkoist brand of environmentalism that prevailed for a time in Russia.

Thus, the focus shifted from the heredity/environment debate to the refutation of the ideological aberrations to which both sides of the controversy were potentially exposed. Worries about the legitimacy of the Resolution were addressed by observing that truth could not be determined by referendum, but that members of scientific societies nonetheless ought to take a position on matters that might affect public policy.

Finally, regarding the ethical question, Statement #2 pointed to the *independence* of moral principles from empirical data, as suggested in the letters:

The harm in the present controversy is not in the suggestion that individuals and groups may differ genetically, but rather in the illogical conclusion sometimes drawn that genetic heterogeneity provides a justification for political and social inequality. It is this inference that we reject and abhor. Political and social equality, and equality of opportunity, are ideals to which we subscribe as citizens; they are not derived from any assumption of biological uniformity. We deplore racism and discrimination not because of scientific knowledge, but because they are contrary to our political values and our respect for humanity. Social policies, including those affecting educational practice, should recognize human diversity by providing the maximum opportunity for the potential of a person to be realized, not as a member of any social or racial group, but as an individual.

Between June and July 1975, Statement #2 circulated among the members of the GSA. Although Smithies’ version of the Resolution met the expectations of the critical side of the Society, it sowed discontent in the side that supported the previous, less impartial version.

### Dissenting Voices on Statement #2

The committee received several letters responding to Statement #2, some of which expressed gratitude for the revisions, others disappointment. Among those who lamented that the committee’s decision process was unfair to the will of the membership were Harrison Echols, Patricia Lawrence, Richard Lewontin, David Perkins, and David Radin. Lewontin, a geneticist and evolutionary biologist at Harvard University who was on the front lines of the IQ controversy (Lewontin 1970, 1974; Rose et al. 1984), disagreed with the decision to rewrite the statement—in his opinion drastically—to respond to a handful of criticisms, despite the overwhelming positive response of the membership to the previous version.^31^ Patricia Lawrence (University of California, Berkeley) described the new procedure as “autocratic,” as it implied that the minority of persons who opposed the previous version possessed some superior qualities that the supporting members lacked.^32^

Several scholars pointed out that the new Resolution confused heritability with inheritance and genetic causality. Evolutionary and molecular biologist Thomas Gregg of Miami University, for example, wrote:

[The Resolution] implies that high heritability means that IQ is mostly determined by genetic factors. It then follows if most geneticists were convinced that IQ has a high heritability, we would be forced to admit that racial differences in IQ were in fact genetically determined. It is absolutely essential for us to clearly point out [that], despite widespread assumptions to the contrary, heritability values alone do not provide information about how important genetic factors are in the determination of a trait or, more importantly, whether there are genetic differences between races.^33^

It is worth considering Gregg’s point more closely. The relationship between heritability and genetic causality has been the subject of intense debates and a common source of misunderstanding underlying the IQ controversy. Indeed, since heritability is a statistical parameter involving variance in populations, many have argued that heritability data (usually acquired through adoption and twin studies) tells us little about the mechanism through which genes influence individual differences in complex phenotypes, or about phenotypic development more generally (see Downes and Matthews 2019; Colman 2016; Gottlieb 1995; Lewontin 1974; Oftedal 2005; Schaffner 2016; Serpico 2018; Tabery 2014; Visscher et al. 2008; Wahlsten 1994).

This point is significant in ethical terms, too, because it reflects concerns about how the public could interpret the genetic data with respect to the problem of human equality. Indeed, the high heritability of a trait is frequently understood as indicating that the development of such trait is strongly determined by genes and thus “fixed,” canalized, or insensible to environmental influences. However, this is a crucial misunderstanding: the high heritability of a trait is compatible with its plasticity (Griffiths 2002; Sauce and Matzel 2018; Serpico and Borghini 2020).^34^ Thus, the target audience of the Resolution was probably not interested in heritability per se, but rather in the canalization or plasticity of intelligence (a question about which the genetics methods available in the 1970s could provide no clear answer). The difficulty of drawing this technical distinction into a statement for public consumption was likely one of the central limitations of the early versions of the Resolution*,* which were partly addressed in the final version.

Other geneticists—for example, Justin Frost, Douglas Futuyma, Sanford Lacks, Patricia Lawrence, Satya Prakash, David Radin, John Turner, and Evelyn Witkin—blamed the committee for having lessened the potential appeal of the Resolution for the public. In their view, while the original statement was a public service, the new one lost sight of the original motivations behind the GSA’s action, namely, persuading geneticists to oppose unwarranted (and potentially racist) interpretations of biological findings. Moreover, the new version was considered too neutral, and thus unhelpful for taking a position on the issue, because it said nothing with which someone could disagree.

Importantly, the correspondence concerning Statement #2 drew attention to the relationship between science and society. David Radin, a geneticist at the University of California, Berkeley, highlighted that geneticists—*as geneticists*—have a responsibility in social matters in which the interpretation and application of their science is at issue.^35^ Justin Frost, also at Berkeley, argued that announcing the results of the poll was by no means an attempt to decide scientific truths by referendum; the issue at stake was not a matter of science but rather one of social responsibility:

If persons like Shockley go around saying there is convincing evidence of genetic difference in intelligence between races the public should be made equally aware that, in the judgement of most geneticists, the present evidence is not convincing. … Ordinarily of course we do not poll ourselves on questions of scientific controversy, such as the best model of chromosome structure, because it is inappropriate for science and public policy is not affected by current judgements on chromosome structure. But in this case scientific judgements about IQ studies are being used in an attempt to influence public policy and the public has the right to be informed about these judgements. The poll is for the use of the public, not the science of the matter.

Frost also openly criticized quantitative genetic methods for their limited value in the study of human behavior:

If the application of quantitative genetics to human traits is fraught with complications and potential biases, why should such research be encouraged? … What is the point of ascertaining IQ equality or non-equality between races if it has no effect on public policy?^36^

This question was also raised by Satya Prakash (a molecular biologist then at the University of Rochester), who saw no valid reason why research in quantitative genetics should not be discouraged.^37^ Relatedly, Futuyma wrote that research on the heredity of group differences “should be done if its results can be envisioned to have substantial social benefit.” He continued:

But it is not clear how generalizations about mean genetic differences among social groups, if proven, would be socially useful, if we subscribe to the ... principle that each individual is to be nurtured and provided with equal opportunity. For the only way in which a generalized statement about a group can be used is by treating each individual (or at least many individuals) in that group in a manner determined by his or her membership in that group, rather than by his or her individual characteristics.^38^

In sum, Smithies’ Resolution was judged as too impartial, and thus unfaithful to the original aims of the GSA’s action. In order to address these criticisms, Smithies made a new motion aimed at deciding whether to publish the first or the second version of the Resolution.

## The Final Version of the Resolution

The decision about the fate of the Resolution was scheduled to be taken during the 44th GSA Annual Meeting in Chapel Hill, North Carolina, in August 1975. Members were to be asked to express their opinion on the two alternative documents. Unfortunately, a definitive decision was not reached, probably because many disagreed with the attempt to make this decision at a meeting that some committee members were unable to attend. As a consequence, Russell was asked to draft yet a third version (hereafter Statement #3) that could bridge the gap between the previous two iterations. In late November 1975, a special meeting took place at O’Hare Airport between the members of the committee, with James Crow (University of Wisconsin-Madison, having replaced David Perkins), along with two additional “non-voting” society members, Douglas Futuyma (SUNY, Stony Brook) and Sewall Wright (University of Wisconsin-Madison), where they were to discuss and improve the final draft.^39^

Essentially, Statement #3 preserved the moderate tones of its immediate precursor. As regards the scientific thesis, the statement invited caution in interpreting genetics data:

Although there is substantial agreement that genetic factors are to some extent responsible for differences in IQ within populations, those who have carefully studied the question disagree on the relative magnitudes of genetic and environmental influences and on how they interact. … In our view, there is no convincing evidence as to whether there is or is not an appreciable genetic difference in intelligence between races.^40^

No connection between hereditarianism and Nazi Germany was drawn, nor between environmentalism and Lysenkoism, making Statement #3 even more impartial. The choice not to mention the political connotations of the debate allowed the GSA to achieve impartiality about scientific opinions, but it left aside important—though difficult—questions.

In this version, the concept was introduced for the first time that the high heritability of a trait does not imply canalization:

Although the variation in a trait may be largely genetic, this does not mean that the degree of expression of that trait cannot be altered by environmental manipulation. Nor does a large environmental component in variation necessarily imply that we can easily change it.

As in Statement #2, the ethical thesis was clearly separated from the scientific one, and it emphasized the loose connection between empirical data and moral principles:

Social policies, including those affecting educational practice, should recognize human diversity by providing the maximum opportunity for all persons to realize their potential, not as members of races or classes but as individuals. We deplore racism and discrimination, not because of any special expertise but because they are contrary to our respect for each human individual. Whether or not there are significant genetic inequalities in no way alters our ideal of political equality, nor justifies racism or discrimination in any form.

Finally, the statement noted that genetic research may yield valid and socially useful results and should not be discouraged, but that it was scientists’ responsibility to speak out against any political misuse of genetics and the drawing of social conclusions from inadequate data.

In January 1976, Statement #3 was circulated to the members of the Society. Most responded positively to the new version: 1,488 members expressed their opinion, with 94 percent supporting the publication of the document. As a result, in July 1976, the final version of the Resolution was finally published, appearing in *Genetics*, volume 83, no. 3, part 1 supplement; it is now available online in the historical records of the Genetics Society of America.^41^ According to Provine, the fact that the statement was “buried” in the Supplement, separated from the scientific journal, contributed to reducing the public resonance of the document: “Most members of the GSA never even knew that the resolution was published and certainly no attempt was made to widely publicize the resolution to the news media as originally intended” (Provine 1986, p. 880).

## The Limited Impact of the Resolution

Although the GSA was and is considered one of the world’s foremost scientific societies, and although the Resolution was approved by hundreds of internationally renowned scholars, it received little attention from both academic and non-academic audiences.^42^ In his publications, Provine suggested that the GSA could have issued a “politically effective statement” that was a clear rebuke to hereditarianism (1979, p. 34; 1986, p. 881). However, the committee eventually realized that the genetics community was far less unanimous on the topic than it had expected. Many scientists defended the reliability of psychometrics and genetics data as well as hereditarianism—in one form or another. It is likely that this made it impossible to reject the hereditarian hypothesis without disappointing the many members asking for more impartiality.

Provine also pointed to scientists’ education as “inadequate” for framing their positions (1979, p. 34). This may have been why GSA members were unable to recognize the moral issue at stake and so supported a Resolution implying that the principle of equal opportunity should depend on the genetic equality of all humans. In other words, the Resolution implied that an *ought* (“we should treat all humans as equal”) could be derived from an *is* (“all humans are genetically equal”). However, there are also good reasons to think that those involved in the discussion were far from being ethically naïve. Indeed, the correspondence shows far more reflectiveness than Provine acknowledged—something that brings to the surface the complexities that probably discouraged stronger political action.

The ethical awareness of the GSA members is testified by the fact that the inference from an *is* to an *ought* was present only in Draft #1, which was not sent to the membership. By contrast, both Statement #2 and Statement #3 deplored racism and discrimination on the basis that they were contrary to shared ethical values.

It was also very clear to the members of the committee that the aim of the GSA’s action was “purely political,” as Turner described it,^43^ because both its motivations and objectives involved political elements. For instance, Turner noted that much of the debate concerned the principle of equal opportunity rather than science, and stressed the importance of not making the mistake that Jensen seemed to be guilty of, namely, imagining that intellectual debates take place in an apolitical vacuum.^44^ Similarly, Futuyma argued that it is not always possible to dissociate the practice of science from the political bases that serve to determine what the applications of the research will be.^45^ Echols noted that it was only through a political statement that the misuse of scientific knowledge could be avoided.^46^ In addition, Turner pointed out that:

one interpretation of the findings seems to support racism, and the natural revulsion for this can be used to drum up feelings in favour of the extreme and doctrinaire environmentalism popular with some psychologists. Geneticists are caught in the middle: we are invited to join irrational environmentalism for fear of seeming to support racism.^47^

These comments clearly denote sensibility and an understanding of the subtleties underlying the debate. It is probably such an awareness that led the committee to try to issue a neutral statement that omitted the political dimension of the debate.

Indeed, the correspondence on this topic revealed a tension between the scientists’ faith in academic freedom and concerns for the potentially harmful implications of the genetic data—especially considering that the data might have turned out to be inaccurate. Given the difficulty of resolving this tension, the only viable option was to disconnect ethical principles and empirical data from each other (a strategy that no members criticized).

An interesting consequence of pursuing impartiality was that the committee was unable to inspect the full range of implications of the stated disconnection between science and ethics. The ethical position expressed in the Resolution involved the following principle: whether or not there are significant genetic inequalities, this in no way alters our ideal of political equality. Thus, social policies should provide equal opportunity for all persons to realize their potential, not as members of groups, but as individuals.

One implication questions the social utility of research on the genetics of group differences. This was suggested by Futuyma when he wrote, “it is not clear how generalizations about mean genetic differences among social groups, if proven, would be socially useful, if we subscribe to the … principle that each individual is to be nurtured and provided with equal opportunity.”^48^ In other words, research on this topic might not be relevant to social policies at all because, regardless of what genetics research will find, society will continue to give equal educational opportunities to all individuals regardless of their background. This implication may not have been accepted by the GSA membership because it directly questioned how worthwhile the whole field of quantitative behavioral genetics was.

Ultimately, the committee realized that it was not possible to write a politically meaningful or effective statement as *scientists*—and certainly not as a *scientific society*. This sheds new light on the “burying” of the Resolution, which was probably not one of the causes of its limited impact, as Provine suggested (1986, p. 880), but rather a *symptom* of the awareness that the committee gained throughout the process.^49^ This is a very natural interpretation of the events if we consider that, despite the political engagement of its members, the committee produced a series of drafts that progressively separated scientific and practical aspects, to the point that political action gave way to a statement recognizing the mere existence of disagreement among scientists on the genetics of group differences.

## What Can the Case of the Resolution Teach Us?

Regarding the trajectory towards impartiality, one may wonder whether the GSA committee could have done anything differently to make the statement more effective or meaningful for the public. In this respect, a comparison of their process with that taken by other scientific societies in the 1960s and 1970s can be instructive.

The GSA was not the only scientific society in this period that was concerned about issues at the intersection between genetics and society. The American Society of Human Genetics (ASHG) contemplated ways to engage with public debates on human populations, psychology, and biology. However, leaders of the Society were worried about achieving a consensus among members, and felt that the Society could not make any public statement in the absence of definitive evidence or unbiased studies on the topic. As Mary Mitchell noted, they were also concerned about professional issues in legitimizing the new discipline (2017).

Eventually, the ASHG appointed a Social Issues Committee for exploring general social aspects relating to genetics, which eventually began to focus on genetic screening and prenatal diagnosis. In its work, the ASHG committee tried to build a wall between matters of fact and matters of value, with the aim of promoting a precise vision of the role of geneticists in society—in Mitchell’s words, “a technocratic advising role and a neutral, apolitical facade” (2017, p. 437).^50^ Thus, in the case of the ASHG’s deliberations, the performance of political neutrality reflected a rather clear political agenda.^51^

By contrast, the GSA’s action was motivated, from the very beginning, by the aspiration of taking an active part in the wider IQ controversy. The correspondence about the issue reveals the aims of informing educators and policymakers, countering racism, and settling the IQ controversy at all levels. In this sense, the pursuit of neutrality seems to reflect no clear political agenda, apart from the mere attempt of achieving a consensus between the society’s members.

Despite the political inclinations of the members of the committee, as well as the pragmatical motivation of the GSA’s action, the wall between facts and values eventually had to be rebuilt. In this sense, the history of the Resolution points to the disconnection between the ultimate goal of settling sensitive debates (which perhaps cannot be other than political) and the ways in which scientific societies are expected to function by their members, the public, and other institutions.^52^ It may be that there was simply no way for the GSA to deliver a message with some practical (social, educational, political) effect. Although a definitive answer to this question cannot be provided here, this is certainly an aspect that contemporary scientific societies may wish to take into consideration in the drafting of statements like the Resolution; it is possible, that is, that taking a political stance at any level is not a viable option for scientific societies.

Another factor involved in the limited impact of the Resolution, for which there was probably little remedy, concerns the way in which the public tends to perceive statements criticizing existing literature rather than developing alternative, positive proposals. As Philip Kitcher noted, criticisms or retractions do not entirely counter the force of the initial announcement and usually do not receive the same publicity (1997, p. 302). It is plausible that the limited impact of the Resolution—as well as of other statements of the same sort—depended on such an asymmetry. By their very nature, statements by scientific societies are often solicited by the publication of other (usually controversial) works.

Given such considerations, in trying to identify potential lessons for the future, it may be useful to focus on the following question: Is there anything that the GSA—*as a scientific society publishing a statement in response to other publications*—could have done differently?

As regards the scientific content, apart from a brief reference to the heritability/canalization distinction, the final Resolution put little emphasis on the role of the environment in shaping human psychological development—an aspect in which the public is usually very interested. The committee could have included this topic in the Resolution without loss of impartiality, as no GSA member ever denied the significance of the interactions between an organism and its environment. This certainly is a technical problem, but still a crucial one that may have an effect on the public’s understanding of the IQ controversy in all its forms.^53^ Even more worrisome is the absence of any discussion as to whether the human groups investigated within genetic research corresponded to “biological categories.” In the Resolution, there is reference to the fact that intellectual abilities are not uniformly distributed in human populations:

All human populations have a vast store of genes in common; yet within populations, individuals differ in genes affecting many characters. Each population contains individuals with abilities far above and below the average of the group.^54^

However, the concept of race itself is never questioned. This is surprising if we consider that the 1950s UNESCO’s discussion (1950, 1952) involved major debates on the validity of the concept, attesting the importance of the problem for scientists of various backgrounds (Brattain 2007).^55^

Overall, given the scientific “inclination” of the final Resolution (at the expense of its political connotation), one would expect more attention paid to cardinal scientific problems like the two above. But what about the relationship between science and politics? It is worth mentioning that the very meaning of the two terms was never explicitly discussed in the correspondence, although many scholars wrote extensively about the ethical, social, and political implications of genetic findings. It is plausible that the writers attempted to keep scientific and political aspects separate from each other in order to avoid the politically loaded overtones that usually characterized the work of scholars who were personally involved in the IQ controversy. This is attested, for instance, by the fact that no member of the committee was inclined to support the hereditarian view, and yet only Echols advocated making a strong statement to this effect (see Provine 1979, p. 23).

As I have shown, the correspondence between the GSA members favored an even stronger disconnection between the ethical and scientific aspects of the controversy. However, by pursuing such a disconnection, the Resolution was unable to uncover ways in which non-scientific values can affect empirical research. For example, whether IQ tests measure intelligence is a technical question concerning construct validity in psychometrics (Cianciolo and Sternberg 2008). However, it also depends on how one defines intelligence and which cognitive abilities are considered relevant for such a trait, and thus involves perspectival, cultural, and social elements (Richardson 2000; Sternberg and Grigorenko 2004). Likewise, the partitioning of humans into distinct groups can depend on pre-scientific, interest-relative, or even biased interpretations of what matters as a group-defining characteristic (for example, skin color, culture, or geographical factors).

A full exploration of the relationship between values and science is beyond the aims of this paper.^56^ However, it should be noted that philosophical debates on this topic in the 1970s were not as developed as they are today (although this is an aspect that Douglas Futuyma, for instance, suggested considering). Contemporary scientific societies certainly have more resources than the GSA did several decades ago.

These considerations suggest that scientific research and findings are neither disconnected from their *motivations,* nor from their *effects*. In a sense, scientific research always involves some political factors, and a clear-cut distinction between science and politics can be elusive (if not misleading) in some contexts. As some GSA members suggested in their correspondence on this topic, the very choice to investigate the relationship between IQ and genetics across human populations may reflect specific political beliefs.

One final aspect that the Resolution could have discussed more is the responsibility of scientists. The document fails to emphasize the essential need to ensure the highest standards of quality, precision, and reliability of scientific research on this topic.^57^ The GSA could also have noted that scientists working in this area have a duty to prove not only that their work will have *positive effects* on society, that is, will help people achieve their full potential (Hunt and Carlson 2007a), but also that empirical findings will have no *negative effects,* that is, will not increase racist feelings and discrimination.^58^ The GSA ought to have taken a clearer and sharper position on this point.

## Conclusion

In this paper, I delineated the three-year process that shaped the “Resolution of Genetics, Race, and Intelligence” published by the Genetics Society of America in 1976. I also outlined the main motivations behind the GSA’s action and discussed the reasons for its failure to achieve its aim of settling the IQ controversy. I made the case that the limited impact of the Resolution mostly depended on the scientific and political impartiality of the final version, which reflected a shift in the aims of the committee due to the extensive controversies that surrounded the GSA’s action. In its early stages, the document was a statement written by scientists for public consumption. At the end of the process, however, the Resolution turned into a report on scientists’ disagreement on the genetics of group differences. The public impact of a statement of this sort is naturally limited in comparison to the impact the earlier versions might have had.

The cautious tones and impartiality made it hard to see the very meaningfulness of the Resolution*.* In retrospect, defending some specific scientific ideas and taking a political stance would probably have been necessary in order to make the statement as impactful as expected. However, as the correspondence between the society’s members attests, many believed that this was outside the capacities of a scientific society like the GSA. This is even more telling if we consider the marked political inclination and sensibility of the members of the committee.

In this sense, the history of the Resolution is particularly enlightening as regards attempts to settle debates at the crossroad of science, ethics, and politics. It is only by looking at the history of the whole affair that we can appreciate the importance of the GSA’s participation—including its failure—in debates on genetics, intelligence, and human populations, and possibly beyond.

## Data Availability

The materials reviewed in this paper are accessible at the University of Leeds Library, Special Collections (MS 2044, see https://explore.library.leeds.ac.uk/special-collections-explore/510877/john_r_g_turner_collection?query=john%20turner&browseQuery=Search&resultOffset=1, particularly MS 2044/1/3/14, box 11) and at the Genetics Society of America Records, American Philosophical Society Library, Philadelphia, Pennsylvania (Mss.575.06.G28p, see https://search.amphilsoc.org/collections/view?docId=ead/Mss.575.06.G28p-ead.xml). The materials archived at the University of Leeds are reproduced with the permission of Special Collections, Leeds University Library. Permission for quoting private letters and documents was kindly provided by the following rights holders: Catherine Crow Rasmussen, Laura Crow, and Frank Crow, descendants of James F. Crow (Professor Emeritus, Laboratory of Genetics, University of Wisconsin, US); Robert G. Fowler (Professor Emeritus, Department of Biological Sciences, San Jose State University, US); Douglas J. Futuyma (Distinguished Professor of Ecology and Evolution, Stony Brook University, US); Thomas G. Gregg (Professor Emeritus, Zoology Department, Miami University, US); Richard C. Lewontin (Alexander Agassiz Professor Emeritus, Harvard University, US); Jack Russell, Jim Russell, and Ellen Gilmore, descendants of Elizabeth S. Russell (The Jackson Laboratory, US); John R. G. Turner (Emeritus Professor, School of Biology, University of Leeds, UK); Evelyn M. Witkin (Barbara McClintock Professor of Genetics, Emerita, Rutgers, the State University of New Jersey, US).

## Footnotes

1 Letter from Evelyn Witkin to Oliver Smithies, 6 September 1975, John R. G. Turner Collection, University of Leeds Library, Special Collections, MS 2044, https://explore.library.leeds.ac.uk/special-collections-explore/510877, Leeds, England; hereafter Turner Collection.

2 Provine’s analysis was based on copies of drafts of the resolution and private exchanges between GSA members that were provided to him; the materials were deposited at the American Philosophical Society in September 1977; see Genetics Society of America Records (Mss.575.06.G28p); Committee on Genetics, Race, Intelligence, 1974–1975, Box 37, American Philosophical Society Library, Philadelphia, Pennsylvania, https://search.amphilsoc.org/collections/view?docId=ead/Mss.575.06.G28p-ead.xml (hereafter GSA Records, APS). According to the cover letter from by David Perkins (president of GSA) to Whitfield J. Bell, Jr. (APS executive director and librarian), 23 September 1977, the material included the records of the committee chair, Elizabeth Russell, as well as additional materials he added. Turner, originally a member of the committee, also provides his own “recollection of the background to the committee and the statement”; see GSA Records, APS, Committee on Genetics, Race, and Intelligence—Background.

3 The material here reviewed (accessed in Spring 2018) is part of a collection of papers that John R. G. Turner donated to the University of Leeds Library, Special Collections and that is currently being processed. For the letters relating to the GSA’s Resolution, see especially MS 2044/1/3/14, box 11.

4 The GSA Records at the APS include early exchanges between members of the Committee on Genetics, Race, Intelligence that are not in the Turner Collection, including the petition for signatures prepared by Harrison Echols; see GSA Records, APS, Committee on Genetics, Race, Intelligence—Background Material, “Petition Concerning Genetics, Race and Intelligence.” https://diglib.amphilsoc.org/islandora/object/text:268358/#page/7/mode/1up. The Turner Collection includes three additional drafts of the Resolution as well as letters and two documents by Douglas Futuyma and John Turner discussed here. It should be noted that, although Futuyma’s and Turner’s documents are not available at the APS archive, Provine (1979) cited them. He probably received them from Russell, chair of the ad hoc Committee, together with a copy of the correspondence between GSA members. See David Perkins to Elizabeth Russell, 11 February 1976, Committee on Genetics, Race, Intelligence – Correspondence.

5 Notably, the files at Leeds include the letters of several persons besides Horowitz who also discuss ethical and political issues, as I will discuss throughout this paper.

6 Jensen drew on twin and adoption studies available at that time (e.g., Burt 1966; Newman et al. 1937; Shields 1962), according to which the heritability of IQ ranges between 0.5 and 0.8; for an overview of these data, see Eysenck and Kamin (1981). A heritability index is a measure of how much the observed variance in a trait (in a specific population, in a specific environment) is statistically associated with genotypic variance. As I explain below, there have been extensive debates about whether heritability data can be interpreted in terms of genetic determination.

7 Shockley’s pronouncements on race and heredity had already begun to cause discontent within the academic community in the mid-1960s, attracting criticism from other Stanford scientists and members of the American Society of Human Genetics (Mitchell 2017) and of the American Association for the Advancement of Science (1967).

8 Douglas Futuyma, “Considerations for the Drafting of a GSA Statement on Race and IQ,” undated, Turner Collection, Leeds.

9 See GSA Records, APS, Committee on Genetics, Race, Intelligence—Background Material, APS, “Petition Concerning Genetics, Race and Intelligence,” https://diglib.amphilsoc.org/islandora/object/committee-genetics-race-intelligence-background-material#page/7/mode/1up. As Provine described, Echols was a member of a small group at Berkeley, known as the Committee on Genetics and Society (CGS), consisting of around fifteen professors, postdocs, and graduate students who had been meeting to “discuss issues of genetics, race and intelligence.” Echols and other CGS members were dismayed that the GSA session did not discuss what they considered to be key questions; hence, their decision to submit a resolution at the GSA Business Meeting (Provine 1976, pp. 21–22).

10 “Proceedings of the 42nd Annual Business Meeting,” GSA Records, Committee on Genetics, Race, Intelligence—Background Material, APS, https://diglib.amphilsoc.org/islandora/object/committee-genetics-race-intelligence-background-material#page/6/mode/1up.

11 On this sequence of events, see also Elizabeth Russell, “Present Status of the Resolution on Genetics, Race and Intelligence prepared by the GSA ad hoc committee”, GSA Records, Committee on Genetics, Race, Intelligence – Draft Resolutions, Folder 1, APS, https://diglib.amphilsoc.org/islandora/object/text:270435#page/3/mode/1up.

12 According to Provine, Russell was selected as chair because she was considered “one of the nicest and most respectable persons alive, so members would naturally support her efforts to produce a resolution” (1979, p. 23).

13 Elizabeth Russell, “Proposed Position Paper – Genetics Society of America,” undated, Turner Collection, Leeds.

14 The committee produced two other drafts, neither of which are in the Leeds or APS archives; however, these two drafts were probably very similar to Draft #4.

15 Elizabeth Russell, “Proposed Position Paper – Genetics Society of America,” undated, Turner Collection, Leeds.

16 I thank two anonymous reviewers for pointing to these questions, to which I will return later. One may wonder whether these questions are exclusively scientific and why the committee kept scientific and political aspects separated from each other.

17 John Turner to Elizabeth Russell, 28 April 1974, Turner Collection, Leeds.

18 Elizabeth Russell, “Genetics Society Resolution on Genetics and IQ – Draft – to be submitted to the ad hoc GSA Committee,” January 1974, Turner Collection, Leeds.

19 For the Resolution drafts held at APS, see https://diglib.amphilsoc.org/islandora/object/text:270435. Discussion of these drafts by committee members and others is available in (digitized) letters in GSA Records, APS: Committee on Genetics, Race, Intelligence—Committee Correspondence, 1974–1975.

20 Elizabeth Russell, “Proposed Position Paper – Genetics Society of America—Draft #4,” undated, Turner Collection, Leeds.

21 Russell laid out the slight differences in Draft #4 from previous versions in a letter to J. Spofford. Letter from Elizabeth Russell to Janice Spofford, 13 May 1974, GSA Records, APS, https://diglib.amphilsoc.org/islandora/object/text:270244/#page/7/mode/1up.

22 Of the four versions of the scientific thesis, the committee adopted the moderate one, according to which existing data on IQ scores of identical twins raised apart convinced some, but not all, geneticists of a considerable degree of heritability of IQ.

23 It is worth noting that the number of responses is remarkable given that all communications between the Society and the members were transmitted via regular mail service.

24 Earl Green to Elizabeth Russell, 28 February 1975, Turner Collection, Leeds.

25 Robert Fowler to Oliver Smithies (president of GSA), 9 September 1975, Turner Collection, Leeds. Although this letter was written later than the others, it discusses the contents of Statement #1.

26 Note that some forms of hereditarianism do not imply strong genetic determinism, but rather hold that genetic factors play *some* role in individual IQ differences. Nonetheless, for many scholars, such a moderate form of hereditarianism is equally unacceptable if based on the analysis of variance or twin and adoption studies; see, for example, Bailey (1997) and Kaplan (2015).

27 Bernard Davis to Oliver Smithies, 4 April 1975, Turner Collection, Leeds.

28 John Turner to Elizabeth Russell, 15 April 1975, Turner Collection, Leeds.

29 Oliver Smithies, “Statement (essentially as presented at GSA Business Meeting, 8/14/1975),” Turner Collection, Leeds. See also Oliver Smithies to GSA Members, 15 July 1975, GSA Records, APS, Committee on Genetics, Race, Intelligence—Draft Resolutions, Folder 1, https://diglib.amphilsoc.org/islandora/object/text:270435#page/12/mode/1up.

30 Oliver Smithies, “Statement of GSA Members (Draft of Revised Version),” 3 July 1975, GSA Records, APS, Genetics, Race, Intelligence Committee on Genetics, Race, Intelligence—Draft Resolutions, Folder 1, https://diglib.amphilsoc.org/islandora/object/text:270435#page/10/mode/1up.

31 Richard Lewontin to Oliver Smithies, 1 August 1975, Turner Collection, Leeds.

32 Patricia Lawrence to Oliver Smithies, 4 August 1975, Turner Collection, Leeds.

33 Thomas Gregg to Oliver Smithies, 30 October 1975, Turner Collection, Leeds.

34 The concept of canalization was used by Conrad Hal Waddington to denote “the property of a developmental process of being to some extent modifiable, but to some extent resistant to modification” (1961, p. 270). Thus, it involves both phenotypic plasticity and robustness: on the one hand, plasticity concerns the ability of environmental influences to alter genetic expression (Bradshaw 1965); on the other hand, robustness concerns the ability of an organism to develop as a typical individual of its species (and thus to have certain phenotypic traits) despite minor perturbations from the genotype and the environment (Palmer 1994).

35 David Radin to Oliver Smithies, 11 August 1975, Turner Collection, Leeds.

36 Justin Frost to Oliver Smithies, 13 August 1975, Turner Collection, Leeds.

37 Satya Prakash to Elizabeth Russell, 23 December 1975, GSA Records, APS, Race, Intelligence Committee on Genetics, Race, Intelligence – Correspondence, https://diglib.amphilsoc.org/islandora/object/satya-prakash-x-elizabeth-russell-correspondence-1975#page/4/mode/2up.

38 Douglas Futuyma, “Considerations for the drafting of a GSA statement on race and IQ,” undated, Turner Collection, Leeds.

39 For more details about this phase, see Provine (1979).

40 Elizabeth Russell, “Statement of GSA Members on Heredity, Race, and IQ,” undated, Turner Collection, Leeds. Many slightly different versions of this document are available at APS; see GSA Records, APS, Committee on Genetics, Race, Intelligence—Draft Resolutions, Folder 2, https://diglib.amphilsoc.org/islandora/object/text:270436#page/1/mode/1up.

41 *An Abridged History of the Genetics Society of America*. Compiled from the Records of the Genetics Society of America and the GENETICS Journal, 1931–2008 by staff member Karen Goodman, Spring 2008, pp. 15–16, http://www2.genetics-gsa.org/pdf/GSAhistoryscrapbook.pdf.

42 Provine (1979, 1986) is not the only scholar at the time who lamented that the Resolution’s impact was very limited. For instance, Mertens (1978, p. 110) commented that the document “has not received the wide distribution and publicity it deserves.” In a personal communication (March 2018), John R. G. Turner noted that the final Resolution was “so oracular that it had, I suspect, very little influence.” Another imperfect but still informative piece of evidence for the limited impact of the Resolution is the scarce number of publications that cite it: to my knowledge, apart from Provine’s two papers, only Kevles (1985), Milkman (1978), and Panofsky (2014) mention it. There certainly may be others, but still, the number is surprisingly small when compared to the much wider influence of publications by Arthur Jensen, Hans Eysenck, and Richard Lewontin, and if we consider the public significance of the debate.

43 John Turner, “Some Propositions,” undated, Turner Collection, Leeds.

44 John Turner to Elizabeth Russell, 26 February 1974, Turner Collection, Leeds.

45 Douglas Futuyma, “Considerations for the drafting of a GSA statement on race and IQ,” undated, Turner Collection, Leeds.

46 Harrison Echols to Elizabeth Russell, 3 March 1975, GSA Records, APS, Committee on Genetics, Race, Intelligence – Correspondence, https://diglib.amphilsoc.org/islandora/object/text:268383/#page/11/mode/1up.

47 John Turner, “Some Propositions,” undated, Turner Collection, Leeds.

48 Douglas Futuyma, “Considerations for the drafting of a GSA statement on race and IQ,” Undated, Turner Collection, Leeds.

49 Regarding the committee’s awareness, some expressed concern that the Resolution could be misunderstood. For instance, Janice Spofford (University of Chicago) noted: “As to wider publicity, … I would like to see us be somewhat selective about what is publicized…. I suspect some wider publicity is going to be necessary inasmuch as what gets released is less likely to be distorted if it is done by us.” Janice Spofford to Elizabeth Russell and Oliver Smithies, 7 April 1975, GSA Records, APS, Committee on Genetics, Race, Intelligence – Correspondence, https://diglib.amphilsoc.org/islandora/object/text:270244/#page/1/mode/1up.

50 Questions concerning the relationship between scientific societies and the public extend beyond the domain of the life sciences. For instance, they relate to the attempt to enhance the awareness of mathematics in the postwar era, in which the American Mathematical Society aimed at taking a public posture to communicate the relevance of mathematics to society (see Barany 2017).

51 As regards the IQ controversy, the ASHG committee invited James Crow (1969) and Luigi Cavalli-Sforza and Walter Bodmer (1970) to write articles challenging Jensen’s arguments, but the Society itself never took a collective position.

52 I thank an anonymous reviewer for suggesting that I explore this aspect further.

53 One might argue that we now have much more evidence on the topic than we had in the 1970s, so that we cannot blame the GSA for not having addressed the problem. However, work on genetic interactions dates back to the early twentieth century; for example, see Bateson (1909); Carlborg and Haley (2004); Cordell (2002). Genotype-environment interactions were well established and discussed with respect to the study of norms of reaction and within discussions on the limitations of quantitative genetics; for example, Dobzhansky (1955); Lewontin (1974); see also Fuller et al. (2005); Nelson (2013); Paul (1998); Serpico (2020).

54 Elizabeth Russell, “Statement of GSA Members on Heredity, Race, and IQ,” undated, Turner Collection, Leeds.

55 For recent statements on this topic from scientific societies, see ASHG (2018), Fuentes et al. (2019). For a critical review on this topic, see James and Burgos (2020).

56 For classical works in this area, see Douglas (2009), Laudan (1984), and Longino (1996).

57 Some scholars have lamented that this requirement is inappropriate. For instance, Gottfredson (2007) argued that it represents an attempt to suppress research that might produce politically-socially challenging results, and that all studies should be held to high standards. Although research quality is an ideal that every researcher should pursue, requiring higher standards in this field may be a cost worth paying if one considers the potential harm of social policies based on inadequate or biased data; see Hunt and Carlson (2007b); Callier and Bonham (2015).

58 For discussions on scientists’ responsibility, see Douglas (2003), Kitcher (1997, 2001).

## References

[CR1] AAAS: American Association for the Advancement of Science (1967). Racial Studies: Academy States Position on Call for New Research. Science.

[CR2] AAAS Committee on Science in the Promotion of Human Welfare (1963). Science and the Race Problem. The Quarterly Review of Biology.

[CR3] Adams, R. 2019. “Cambridge College Sacks Researcher over Links with Far Right,” 1 May 2019. *The Guardian*, https://www.theguardian.com/education/2019/may/01/cambridge-university-college-dismisses-researcher-far-right-links-noah-carl.

[CR4] APA: American Psychological Association. 2001. *Resolution Against Racism and in Support of the Goals of the 2001 UN World,* https://www.apa.org/about/policy/racism.

[CR5] ASHG: American Society of Human Genetics (2018). ASHG Denounces Attempts to Link Genetics and Racial Supremacy. American Journal of Human Genetics.

[CR6] Bailey RC (1997). Hereditarian Scientific Fallacies. Genetica.

[CR7] Bangham J (2015). What Is Race? UNESCO, Mass Communication and Human Genetics in the Early 1950s. History of the Human Sciences.

[CR8] Barany MJ (2017). The World War II Origins of Mathematics Awareness. Notices of the American Mathematical Society.

[CR9] Bateson W (1909). Mendel’s Principles of Heredity.

[CR10] Bliss C (2018). Social by Nature: The Oromise and Peril of Sociogenomics.

[CR11] Block N (1995). How Heritability Misleads About Race. Cognition.

[CR12] Bradshaw AD (1965). Evolutionary Significance of Phenotypic Plasticity in Plants. Advances in Genetics.

[CR13] Brattain M (2007). Race, Racism, and Antiracism: UNESCO and the Politics of Presenting Science to the Postwar Public. The American Historical Review.

[CR14] Burt C (1966). The Genetic Determination of Differences in Intelligence: A Study of Monozygotic Twins Reared Together and Apart. British Journal of Psychology.

[CR15] Callier SL, Bonham VL (2015). Taking a Stand: The Genetics Community's Responsibility for Intelligence Research. Hastings Center Report.

[CR16] Carlborg O, Haley CS (2004). Epistasis: Too Often Neglected in Complex Trait Studies?. Nature Reviews. Genetics.

[CR17] Cavalli-Sforza LL, Bodmer WF (1970). Intelligence and Race. Scientific American.

[CR18] Cianciolo AT, Sternberg RJ (2008). Intelligence: A Brief History.

[CR19] Colman AM (2016). Race Differences in IQ: Hans Eysenck’s Contribution to the Debate in the Light of Subsequent Research. Personality and Individual Differences.

[CR20] Cordell HJ (2002). Epistasis: What It Means, What It Doesn’t Mean, and Statistical Methods to Detect It in Humans. Human Molecular Genetics.

[CR21] Crow J (1969). Genetic Theories and Influences: Comments on the Value of Diversity. Harvard Educational Review.

[CR22] Cubelli, R. 2010. *Comunicato Stampa, Associazione Italiana di Psicologia*, https://www.aipass.org/sites/default/files/COMUNICATO%20STAMPA%20QI.doc.

[CR23] Daniels N (1973). The Smart White Man's Burden: An Analysis of the Proposition That Poor Blacks Are Polluting the National Gene Pool. Harper’s Magazine.

[CR24] DeFries JC, Ehrman L, Omenn GS, Caspari E (1972). Reply to Professor Jensen. Genetics, Environment, and Behavior: Implications for Educational Policy.

[CR25] Devlin B, Fienberg SE, Resnick DP, Roeder K (1997). Intelligence, Genes, and Success: Scientists Respond to The Bell Curve.

[CR26] Dobzhansky T (1955). Evolution, Genetics, and Man.

[CR27] Douglas HE (2009). Science, Policy, and the Value-free Ideal.

[CR28] Douglas HE (2003). The Moral Responsibilities of Scientists (Tensions Between Autonomy and Responsibility). American Philosophical Quarterly.

[CR29] Downes, S. M. & Matthews, L. 2019. Heritability. In E. N. Zalta (Ed.) *The Stanford Encyclopedia of Philosophy*, https://plato.stanford.edu/archives/win2019/entries/heredity.

[CR30] Eysenck HJ (1971). Race, Intelligence and Education.

[CR31] Eysenck HJ, Kamin LJ (1981). Intelligence: The Battle for the Mind.

[CR32] Flew A (1973). The Jensen Uproar. Philosophy.

[CR33] Fuentes A, Ackermann RR, Athreya S (2019). AAPA Statement on Race and Racism. American Journal of Physical Anthropology.

[CR34] Fuller T, Sarkar S, Crews D (2005). The Use of Norms of Reaction to Analyze Genotypic and Environmental Influences on Behavior in Mice and Rats. Neuroscience &amp; Biobehavioral Reviews.

[CR35] Gottfredson LS (2007). Applying Double Standards to “Divisive” Ideas: Commentary on Hunt and Carlson (2007). Perspectives on Psychological Science.

[CR36] Gottlieb G (1995). Some Conceptual Deficiencies in ‘Developmental’ Behavior Genetics. Human Development.

[CR37] Gould SJ (1981). The Mismeasure of Man.

[CR38] Griffiths PE (2002). What is Innateness?. The Monist.

[CR39] Herrnstein, R. J. 1971. IQ. *Atlantic Monthly* (September), pp. 43–64.

[CR40] Herrnstein RJ, Murray C (1994). The Bell Curve: Intelligence and Class Structure in American Life.

[CR41] Hirsch J (1975). Jensenism: The Bankruptcy of “Science” Without Scholarship 1. Educational Theory.

[CR42] Hunt E, Carlson J (2007). Considerations Relating to the Study of Group Differences in Intelligence. Perspectives on Psychological Science.

[CR43] Hunt E, Carlson J (2007). Reply to Commentators. Perspectives on Psychological Science.

[CR44] James, M., & A. Burgos. 2020. “Race.” In *The Stanford Encyclopedia of Philosophy*, ed. E. N. Zalta, https://plato.stanford.edu/archives/sum2020/entries/race.

[CR45] Jensen AR (1969). How Much Can We Boost IQ and Scholastic Achievement. Harvard Educational Review.

[CR46] Jensen AR (1972). Genetics and Education.

[CR47] Kamin LJ (1974). The Science and Politics of I.Q.

[CR48] Kaplan JM (2015). Race, IQ, and the Search for Statistical Signals Associated With So-called ‘X’-factors: Environments, Racism, and the ‘Hereditarian Hypothesis.’. Biology &amp; Philosophy.

[CR49] Kevles DJ (1985). In the Name of Eugenics: Genetics and the Uses of Human Heredity.

[CR50] Kitcher P (1997). An Argument About Free Inquiry. Noûs.

[CR51] Kitcher P (2001). Science, Truth, and Democracy.

[CR52] Lally, C. 2018. Cambridge Dons Revolt Over ‘Racist’ Fellow’s Role. *The Times*, https://www.thetimes.co.uk/article/cambridge-dons-revolt-over-racist-fellow-s-role-3gfnjrjsh.

[CR53] Laudan L (1984). Science and Values: The Aims of Science and Their Role in Scientific Debate.

[CR54] Levin M (1997). Why Race Matters. Race Differences and What They Mean.

[CR55] Lewontin RC (1970). Race and Intelligence. Bulletin of the Atomic Scientists.

[CR56] Lewontin RC (1974). Annotation: The Analysis of Variance and the Analysis of Causes. American Journal of Human Genetics.

[CR57] Longino HE, Nelson LN, Nelson J (1996). Cognitive and Non-cognitive Values in Science: Rethinking the Dichotomy. Feminism, Science, and the Philosophy of Science.

[CR58] Lynn R (2008). The Global Bell Curve: Race, IQ, and Inequality Worldwide.

[CR59] Lynn R (2010). In Italy, North-South Differences in IQ Predict Differences in Income, Education, Infant mortality, Stature, and Literacy. Intelligence.

[CR60] Meredith, R. 2018. “Calls to Revoke ‘Sexist’ Ulster University Professor.” *BBC News,* https://www.bbc.co.uk/news/uk-northern-ireland-43043242.

[CR61] Mertens TR (1978). Genetic Engineering and Genetics Education. The American Biology Teacher.

[CR62] Milkman R (1978). A Simple Exposition of Jensen’s Error. Journal of Educational Statistics.

[CR63] Mitchell MX (2017). Screening Out Controversy: Human Genetics, Emerging Techniques of Diagnosis, and the Origins of the Social Issues Committee of the American Society of Human Genetics, 1964–1973. Journal of the History of Biology.

[CR64] Nelson RM, Pettersson ME, Carlborg O (2013). A Century After Fisher: Time for a New Paradigm in Quantitative Genetics. Trends in Genetics.

[CR65] Newman H, Freeman F, Holzinger K (1937). Twins: A Study of Heredity and Environment.

[CR66] Oftedal G (2005). Heritability and Genetic Causation. Philosophy of Science.

[CR67] Page EB (1972). Resolution on Scientific Freedom Regarding Human Behaviour and Heredity. American Psychologist.

[CR68] Palmer AR, Markon TA (1994). Fluctuating Asymmetry Analyses: A Primer. Developmental Instability: Its Origins and Evolutionary Implications.

[CR69] Panofsky A (2014). Misbehaving Science: Controversy and the Development of Behavior Genetics.

[CR70] Paul DB (1998). The Politics of Heredity: Essays on Eugenics, Biomedicine, and the Nature-Nurture Debate.

[CR71] Provine WB (1979). Genetics, Race, and Intelligence: The Genetics Society of America Resolution. Cornell Review.

[CR72] Provine WB (1986). Geneticists and Race. American Zoologist.

[CR73] Richardson K (2000). The Making of Intelligence.

[CR74] Rose S, Kamin LJ, Lewontin RC (1984). Not in Our Genes: Biology, Ideology and Human Nature.

[CR75] Rushton J. P. (1995). Race, Evolution and Behavior: A Life History Perspective.

[CR76] Russell ES (1976). Resolution on Genetics, Race, and Intelligence. Genetics.

[CR77] Sauce B, Matzel LD (2018). The Paradox of Intelligence: Heritability and Malleability Coexist in Hidden Gene-Environment Interplay. Psychological Bulletin.

[CR78] Schaffner KF (2016). Behaving: What's Genetic, What's Not, and Why Should We Care?.

[CR79] Serpico D (2018). What Kind of Kind Is Intelligence?. Philosophical Psychology.

[CR80] Serpico D (2020). Beyond Quantitative and Qualitative Traits: Three Telling Cases in the Life Sciences. Biology & Philosophy.

[CR81] Serpico D, Borghini A (2020). From Obesity to Energy Metabolism: Ontological Perspectives on the Metrics of Human Bodies. Topoi.

[CR82] Shields J (1962). Monozygotic Twins: Brought Up Apart and Brought Up Together: An Investigation into the Genetic and Environmental Causes of Variation in Personality.

[CR83] Snyderman M, Rothman S (1988). The IQ Controversy, the Media and Public Policy.

[CR84] Sternberg RJ, Grigorenko EL (2004). Intelligence and culture: how culture shapes what intelligence means, and the implications for a science of well-being. Philosophical Transactions of the Royal Society of London. Series b: Biological Sciences.

[CR85] Tabery J (2014). Beyond Versus: The Struggle to Understand the Interaction of Nature and Nurture.

[CR86] Terman LM (1916). The Measurement of Intelligence: An Explanation of and a Complete Guide for the Use of the Stanford Revision and Extension of the Binet-Simon Intelligence Scale.

[CR87] Turner JRG (1970). Scientific Heresy. Nature.

[CR88] UNESCO: United Nations Educational, Scientific and Cultural Organization (1950). The Race Question.

[CR89] UNESCO: United Nations Educational, Scientific and Cultural Organization (1952). The Race Concept: Results of An Inquiry.

[CR90] Letters: “A Troublesome Inheritance.” 2014. [Response of 143 population geneticists to the review of Nicholas Wade, “A Troublesome Inheritance: Genes, Race and Human History” (July 13)], *The New York Times Book Review,* August 8, 2014.] Stanford Center for Computational, Evolutionary and Human Genomics. https://cehg.stanford.edu/letter-from-population-geneticists.

[CR91] Vetta A (1973). Amendment to the “Resolution on Scientific Freedom Regarding Human Behavior and Heredity”. American Psychologist.

[CR92] Visscher PM, Hill WG, Wray NR (2008). Heritability in the Genomics Era—Concepts and Misconceptions. Nature Reviews Genetics.

[CR93] Waddington CH (1961). Genetic Assimilation. Advances in Genetics.

[CR94] Wade N (2014). A Troublesome Inheritance: Genes, Race and Human History.

[CR95] Wahlsten D (1994). The Intelligence of Heritability. Canadian Psychology.

[CR96] Weinberg, J. 2020. “Scholars Object to Publication of Paper Defending Race Science.” *Daily Nous*, 20 January 2020. https://dailynous.com/2020/01/20/scholars-object-publication-paper-defending-race-science.

